# Associations between triglyceride-glucose index and different hypertension subtypes: A population-based study in China

**DOI:** 10.3389/fcvm.2022.901180

**Published:** 2022-08-11

**Authors:** Qian Cai, Cathleen Y. Xing, Jiang Zhu, Ying Wang, Fanghong Lu, Jie Peng

**Affiliations:** ^1^Department of Geriatric Medicine, Qilu Hospital of Shandong University, Jinan, China; ^2^Key Laboratory of Cardiovascular Proteomics of Shandong Province, Qilu Hospital of Shandong University, Jinan, China; ^3^Tuberculosis Control and Prevention Program, San Francisco Department of Public Health, San Francisco, CA, United States; ^4^Department of Breast Surgery, General Surgery, Qilu Hospital of Shandong University, Jinan, China; ^5^The Key Laboratory of Cardiovascular Remodeling and Function Research, Chinese Ministry of Education, Chinese National Health Commission and Chinese Academy of Medical Sciences, The State and Shandong Province Joint Key Laboratory of Translational Cardiovascular Medicine, Department of Cardiology, Cheeloo College of Medicine, Qilu Hospital of Shandong University, Jinan, China; ^6^Cardio-Cerebrovascular Control and Research Center, Institute of Basic Medicine, Shandong First Medical University and Shandong Academy of Medical Sciences, Jinan, China

**Keywords:** TyG index, hypertension, cross-sectional studies, ISH, IDH, SDH

## Abstract

**Background:**

Abnormal glycolipid metabolism plays a crucial role in hypertension. While an elevated triglyceride-glucose (TyG) index has been recognized as a risk factor for developing hypertension, the associations between the TyG index and different hypertension subtypes, namely, isolated systolic hypertension (ISH), isolated diastolic hypertension (IDH), and systolic-diastolic hypertension (SDH), remain unclear. This study was designed to investigate the associations between the TyG index and hypertension subtypes in a general Chinese population.

**Materials and methods:**

In a sample of 16,793 participants from Shandong Province, China, multivariate logistic regression analyses were performed to examine the associations between the TyG index and different hypertension subtypes. Loess smooth curves were fitted to visualize the trends. Stratified analyses were conducted to further assess the potential interactions in the associations between the TyG index and different hypertension subtypes.

**Results:**

A higher TyG index was associated with an increased odds of having IDH (OR = 2.94, 95% CI: 1.66–5.23) and SDH (OR = 1.82, 95% CI: 1.33–2.49), whereas no apparent relationship was observed between TyG index and ISH. With respect to sex, the effect of TyG index on having IDH and SDH was significant in women, but not in men. Participants with lower lipid profiles and glucose levels demonstrated a stronger strength of association between the TyG index and IDH as compared with the TyG index-SDH association. Stratified analysis showed that participants with a higher TyG index were more than 3 times more likely to have IDH and SDH among persons aged 18–42 years. Significant interactions were observed between TyG index and sex, age, and high-density lipoprotein cholesterol (HDL-C) in the SDH group, and a significant interaction was also found between TyG index and body mass index (BMI) in the ISH group.

**Conclusion:**

Triglyceride-glucose index may potentially serve as a novel indicator for IDH and SDH. Our findings could also inform the development and implementation of targeted screening for hypertension.

## Introduction

Hypertension is a major risk factor for developing cardiovascular disease (CVD) and is increasingly recognized as a serious, worldwide public health concern (1). The prevalence of hypertension is increasing at an alarming rate annually (2). The population in China accounts for 20% of the world’s population and therefore could represent some of the excess burden of hypertension (3). A large national survey from 2012 to 2015 indicated that approximately 244.5 million Chinese adults were diagnosed with hypertension (4); however, the management of hypertension remains largely unsatisfactory (5).

To date, research has demonstrated the associations between CVD and three hypertension subtypes, namely, isolated systolic hypertension (ISH), isolated diastolic hypertension (IDH), and systolic-diastolic hypertension (SDH) (6). It is thought that ISH is related to increased arterial stiffness, and in contrast, IDH is correlated with elevated vascular resistance in the arteriolar sector (7). IDH is more prevalent among young and middle-aged adults, while ISH is more prevalent in the elderly (8). As people age, the elastic fibers of the arterial wall decrease while the collagen fibers increase, leading to stiffness, reduced vascular compliance, and weakened buffering effect on blood pressure (BP). This mechanism may potentially lower diastolic blood pressure (DBP) and further elevate systolic blood pressure (SBP). As a result, some IDH patients tend to develop ISH or SDH as they age (9). Current literature suggests that IDH can be attributed to temporary or long-term lesions of arterioles in the case of good elasticity of large arteries. These lesions could be associated with cigarette smoking, excessive alcohol consumption, obesity, high uric acid, abnormal glucose tolerance, and increased sympathetic activity (10). Hence, identifying potential risk factors for IDH and ISH would be beneficial in understanding the underlying mechanisms and determining more effective treatment plans.

Previous studies have demonstrated that abnormal glycolipid metabolism might be associated with the development of hypertension (11, 12). Metabolic syndromes, especially elevated blood glucose (BG), abnormal plasma triglyceride (TG), elevated total cholesterol (TC), increased low-density lipoprotein cholesterol (LDL-C), and reduced high-density lipoprotein cholesterol (HDL-C), are all considered as potential risk factors for CVD and all-cause mortality (13, 14). Likewise, insulin resistance (IR) plays a key role in the pathogenesis of hypertension (15). Due to the complexity and inaccessibility of performing insulin and C-peptide release assays, a simple indicator that recognizes early IR would be beneficial for substantial clinical hypertension management. In addition, it is noteworthy to point out that while screening for hypertension, clinicians should not only focus on patients with high BG and TG but also focus on patients with normal or borderline BG and TG. For this reason, researchers have proposed the use of triglyceride-glucose (TyG) index, a surrogate indicator of combined serum TG and glucose, to assess the relationship between IR and hypertension in different populations (16).

While an elevated TyG index is a potentially important predictor of hypertension, the association between the TyG index and each hypertension subtype has not been well clarified. Therefore, the purpose of this study is to examine the associations between the TyG index and different hypertension subtypes. Understanding the association between the TyG index and each hypertension subtype is of particular interest due to the importance of determining the impact of glycolipid metabolic disorder on ISH, IDH, and SDH, interpreting the pathogenesis of each hypertension subtype and ultimately optimizing the preventive and treatment strategies.

## Materials and methods

### Study subjects

This study assessed a total of 21,670 participants from a population-based study conducted in Shandong, China, from January 2007 to December 2010. All participants were recruited from local communities, and written informed consent was obtained from each participant before data collection. This study was approved by Shandong First Medical University and Shandong Academy of Medical Sciences and was conducted in accordance with the Declaration of Helsinki.

Given that antihypertensive drugs could significantly lower BP readings, hypertensive participants on antihypertensive drugs could be misclassified as normotensive, if their BP readings measured were all within normal limits. More importantly, antihypertensive drugs such as diuretics (17) and angiotensin II receptor blocker (ARB) (18) may affect glucolipid metabolism. Therefore, inclusion criteria for participants were as follows: (1) ages 18–75 years; (2) with no history of using antihypertensives drugs or had used antihypertensive drugs but had not taken antihypertensive drugs within the past 2 weeks, and (3) with no history of using lipid-lowering drugs (e.g., statins) and/or medications to treat diabetes. Participants who failed to meet the inclusion criteria, as well as participants with secondary hypertension, severe heart failure, renal failure, and/or valvular heart disease were excluded from this study. In total, 16,793 participants were recruited.

### Clinical and biological data collection

Clinical data used in this study were collected through an in-person, clinician-administered questionnaire, which included sociodemographic, lifestyle and behavior, medical history, and use of medication. The vital signs and anthropometric measurements include height, weight, waist circumference, BP, and pulse rate (at rest for 60 s) were noted. Venous blood samples were collected from participants after fasting for at least 12 h for biochemical determination, and participants who did not fast for at least 12 h were asked to reschedule their appointments. Fasting blood glucose (FBG) was measured by the hexokinase method using an autoanalyzer. Similarly, other biomarkers such as TG, TC, HDL-C, and LDL-C were measured by a fully automated chemiluminescence immunoassay. According to the Chinese Guidelines for the Prevention of CVD (v2011), abdominal obesity was defined as (1) waist circumference > 90 cm in men and (2) waist circumference > 85 cm in women. With respect to tobacco use, a participant could be characterized as either a never smoker (e.g., who has never smoked or who has smoked less than 100 cigarettes during lifetime) or a smoker (e.g., who has smoked at least 100 cigarettes during lifetime) (19). Moreover, binge drinking was defined as consuming 5 or more alcoholic drinks for men or 4 or more alcoholic drinks for women on 1 or more days in the past month (20). Body mass index (BMI) was calculated as weight (kg)/[height (m)^2^], and lastly, TyG index was defined as ln [FBG (mg/dl) * TG (mg/dl)/2] (21).

The BP readings were taken using the OMRON HEM-7011 electronic sphygmomanometer (Omron, Japan). According to the 2018 ESC/ESH Guidelines for the management of arterial hypertension (22), BP readings were measured in a seated position after at least 5 min of rest in the morning, with arms placed at the heart level. A total of three BP measurements were taken on the same day, 1–2 min apart, and additional measurements were required only if the first two readings differed by more than 10 mmHg. The final BP was then recorded as the average of the last two BP readings, and IDH, ISH, and SDH were identified. Specifically, IDH was defined as SBP < 140 mmHg and DBP ≥ 90 mmHg, ISH was defined as SBP ≥ 140 mmHg and DBP < 90 mmHg, SDH was defined as SBP ≥ 140 mmHg and DBP ≥ 90 mmHg, and normotension (NM) was characterized as SBP < 140 mmHg and DBP < 90 mmHg. Participants who were previously told to have hypertension at some point but failed to meet our diagnostic criteria were all considered as normotensive (e.g., no ISH, IDH, and SDH) in this study.

### Data analysis

All analyses were performed using R (The R Foundation, version 3.4.3^1^) and Empower^®^ (^2^ Boston, MA, United States). Descriptive statistics were calculated to describe the sociodemographics, BP readings, lipid profiles, anthropometric measurements, smoking status, and binge drinking. Mean values with standard deviations were reported for continuous variables, and frequencies and proportions were presented to describe all categorical variables. *Post hoc* pairwise comparisons with Bonferroni corrections were performed using ANOVA test for continuous variables and χ^2^ test for categorical variables. To examine the relationships between the TyG index and hypertension subtypes, three logistic regression models were built to calculate odds ratios (OR) and 95% confident intervals (CI). In particular, Model 0 was a univariate or unadjusted model, and Model 1 was a multivariate model adjusted for age and sex only. Multivariable Model 2 was controlled for age, sex, pulse, TC, TG, FBG, HDL-C, LDL-C, BMI, abdominal obesity, smoking history, and binge drinking. Smooth curves were applied to illustrate the trends between the TyG index and the predicted probabilities of ISH, IDH, and SDH. Ultimately, stratified analyses were performed to further assess the associations between the TyG index and the continuous variables using quartiles, and interactions between the TyG index and strata variables were tested and reported. All reported *p*-Values were two-sided, and *p* < 0.05 was considered statistically significant.

## Results

### Demographics, lifestyles, and clinical characteristics of study participants

The distribution of characteristics among all participants is shown in Table 1. The mean ages in the ISH and SDH groups were similar (59.03 ± 10.39 years and 54.79 ± 10.39 years), but were significantly higher than the mean ages in the IDH and NM groups (47.53 ± 9.70 years and 48.33 ± 12.02 years), respectively. An increasing trend was observed with regard to SBP readings, DBP readings, and BMI. The mean values of the TyG index, the proportions of participants who smoked at least 100 cigarettes during lifetime, and the proportions of binge drinkers were remarkably higher among participants in the IDH and SDH groups compared with participants in the ISH and NM groups.

**TABLE 1:** Sociodemographics, lifestyles and clinical characteristics, overall and by hypertension subtypes, Shandong, China (2007–2010).

	Overall (*n* = 16,793)		NM (*n* = 9,589)	ISH (*n* = 2,752)	IDH (*n* = 927)	SDH (*n* = 3,525)	*P*-value (overall)
**Continuous Variables, mean ± standard deviation**							
Age (years)	51.40 ± 12.09		48.33 ± 12.02	59.03 ± 10.39*	47.53 ± 9.70#	54.79 ± 10.39*#†	**<0.001**
SBP (mmHg)	136.12 ± 21.51		121.99 ± 10.42	153.02 ± 12.76*	132.22 ± 5.69*#	162.42 ± 17.62*#†	**<0.001**
DBP (mmHg)	83.04 ± 11.08		76.74 ± 6.98	81.66 ± 5.92*	93.77 ± 3.62*#	98.42 ± 6.74*#†	**<0.001**
Pulse (per 60 s)	74.80 ± 9.46		74.21 ± 9.21	73.99 ± 9.65	78.01 ± 9.39*#	76.22 ± 9.67*#†	**<0.001**
TC (mmol/L)	4.63 ± 1.00		4.56 ± 0.98	4.74 ± 1.02*	4.65 ± 1.03	4.71 ± 1.02*	**<0.001**
TG (mmol/L)	1.44 ± 1.10		1.34 ± 1.00	1.48 ± 1.14*	1.66 ± 1.23*#	1.63 ± 1.25*#	**<0.001**
FBG (mmol/L)	5.03 ± 1.44		4.93 ± 1.36	5.21 ± 1.65*	5.06 ± 1.43#	5.15 ± 1.47*	**<0.001**
HDL-C (mmo1/L)	1.57 ± 0.46		1.54 ± 0.45	1.62 ± 0.47*	1.56 ± 0.45#	1.60 ± 0.45*	**<0.001**
LDL-C (mmol/L)	3.05 ± 0.75		3.02 ± 0.74	3.11 ± 0.74*	3.09 ± 0.77*	3.10 ± 0.77*	**<0.001**
TyG index	4.62 ± 0.35		4.58 ± 0.33	4.65 ± 0.35*	4.70 ± 0.37*#	4.70 ± 0.37*#	**<0.001**
BMI (kg/m^2^)	24.30 ± 3.58		23.69 ± 3.36	24.48 ± 3.61*	25.41 ± 3.37*#	25.52 ± 3.77*#	**<0.001**
**Categorical Variables, n (%)**							
Sex							**<0.001**
Male		6,444 (38.37%)	3,392 (35.37%)	1,007 (36.59%)	421 (45.42%)*#	1,624 (46.07%)*#	
Female		10,349 (61.63%)	6,197 (64.63%)	1,745 (63.41%)	506 (54.58%)	1,901 (53.93%)	
Abdominal obesity							**<0.001**
No		9,406 (56.01%)	6,001 (62.58%)	1,380 (50.15%)*	450 (48.54%)*	1,575 (44.68%)*#†	
Yes		7,387 (43.99%)	3,588 (37.42%)	1,372 (49.85%)	477 (51.46%)	1,950 (55.32%)	
Binge drinking							**<0.001**
No		13,837 (82.40%)	8,114 (84.62%)	2,330 (84.67%)	705 (76.05%)*#	2,688 (76.26%)*#	
Yes		2,956 (17.60%)	1,475 (15.38%)	422 (15.33%)	222 (23.95%)	837 (23.74%)	
Smoking history							**<0.001**
Never smoker		13,998 (83.36%)	8,027 (83.71%)	2,352 (85.47%)*	751 (81.01%)*#	2,868 (81.36%)*#	
Smoker		2,795 (16.64%)	1,562 (16.29%)	400 (14.53%)	176 (18.99%)	657 (18.64%)	

Overall p-values generated using one-way ANOVA tests (continuous variables) or Pearson’s chi-square tests (categorical variables). Bold values indicate statistical significance. *p < 0.05 compared with NM, ^#^*p* < 0.05 compared with ISH, and †*p* < 0.05 compared with IDH. BMI, body mass index; NM, normotension; IDH, isolated diastolic hypertension; ISH, isolated systolic hypertension; SDH, systolic-diastolic hypertension; SBP, systolic blood pressure; DBP, diastolic blood pressure; FBG, fasting blood glucose; TG, triglyceride; TC, total cholesterol; LDL-C, low-density lipoprotein cholesterol; HDL-C, high-density lipoprotein cholesterol; TyG index, triglyceride-glucose index.

### Associations between the triglyceride-glucose index and hypertension subtypes

Table 2 shows the univariable associations of TyG index, age, sex, pulse, TC, TG, FBG, HDL-C, LDL-C, BMI, abdominal obesity, smoking history, and binge drinking with hypertension subtypes. Significant positive associations were found between the TyG index and all 3 hypertension subtypes (ISH: OR = 1.31, 95% CI: 1.17, 1.46; IDH: OR = 1.84, 95% CI: 1.55, 2.18; SDH: OR = 2.06, 95% CI: 1.86, 2.27). In addition to the TyG index, all other variables were also found to be significantly associated with ISH and SDH; however, no significant associations of IDH with TC, FBG, HDL-C, and LDL-C were observed. As expected, significant inverse relationships between the TyG index and all co-variables except smoking in the NM group were observed.

**TABLE 2:** Univariate logistic regression analyses of the associations between sociodemographic, clinical characteristics and lifestyles, and hypertension subtypes, Shandong, China (2007–2010).

Variables	NM	ISH	IDH	SDH
	OR (95% CI)	OR (95% CI)	OR (95% CI)	OR (95% CI)
Age (years)	0.95 (0.95, 0.95) ***p* < 0.0001**	1.08 (1.07, 1.08) ***p* < 0.0001**	0.97 (0.97, 0.98) ***p* < 0.0001**	1.03 (1.03, 1.03) ***p* < 0.0001**
Sex (%)				
Male	1.00	1.00	1.00	1.00
Female	1.34 (1.26, 1.43) ***p* < 0.0001**	1.10 (1.01, 1.19) ***p* = 0.0356**	0.74 (0.64, 0.84) ***p* < 0.0001**	0.67 (0.62, 0.72) ***p* < 0.0001**
Pulse (per 60 s)	0.98 (0.98, 0.99) ***p* < 0.0001**	0.99 (0.98, 0.99) ***p* < 0.0001**	1.04 (1.03, 1.04) ***p* < 0.0001**	1.02 (1.02, 1.02) ***p* < 0.0001**
TC (mmol/L)	0.86 (0.84, 0.89) ***p* < 0.0001**	1.13 (1.09, 1.18) ***p* < 0.0001**	1.03 (0.96, 1.10) *p* = 0.4403	1.10 (1.06, 1.14) ***p* < 0.0001**
TG (mmol/L)	0.81 (0.78, 0.83) ***p* < 0.0001**	1.04 (1.00, 1.07) ***p* = 0.0388**	1.14 (1.09, 1.19) ***p* < 0.0001**	1.18 (1.15, 1.22) ***p* < 0.0001**
FBG (mmol/L)	0.90 (0.88, 0.92) ***p* < 0.0001**	1.09 (1.06, 1.12) ***p* < 0.0001**	1.01 (0.97, 1.06) *p* = 0.5246	1.07 (1.04, 1.09) ***p* < 0.0001**
HDL-C (mmo1/L)	0.76 (0.71, 0.81) ***p* < 0.0001**	1.31 (1.20, 1.43) ***p* < 0.0001**	0.96 (0.83, 1.12) *p* = 0.6310	1.20 (1.11, 1.30) ***p* < 0.0001**
LDL-C (mmol/L)	0.86 (0.82, 0.89) ***p* < 0.0001**	1.13 (1.07, 1.19) ***p* < 0.0001**	1.07 (0.98, 1.16) *p* = 0.1494	1.11 (1.06, 1.17) ***p* < 0.0001**
BMI (kg/m^2^)	0.89 (0.88, 0.90) ***p* < 0.0001**	1.02 (1.01, 1.03) ***p* = 0.0033**	1.09 (1.07, 1.11) ***p* < 0.0001**	1.13 (1.11, 1.14) ***p* < 0.0001**
TyG index	0.43 (0.40, 0.48) ***p* < 0.0001**	1.31 (1.17, 1.46) ***p* < 0.0001**	1.84 (1.55, 2.18) ***p* < 0.0001**	2.06 (1.86, 2.27) ***p* < 0.0001**
Abdominal obesity				
No (referent)	1.00	1.00	1.00	1.00
Yes	0.54 (0.50, 0.57) ***p* < 0.0001**	1.33 (1.22, 1.44) ***p* < 0.0001**	1.37 (1.20, 1.57) ***p* < 0.0001**	1.78 (1.65, 1.92) ***p* < 0.0001**
Smoking history				
Never smoker (referent)	1.00	1.00	1.00	1.00
Smoker	0.94 (0.87, 1.02) *p* = 0.1550	0.83 (0.74, 0.93) ***p* = 0.0012**	1.19 (1.00, 1.40) ***p* = 0.0491**	1.19 (1.08, 1.31) ***p* = 0.0004**
Binge drinking				
No (referent)	1.00	1.00	1.00	1.00
Yes	0.70 (0.65, 0.76) ***p* < 0.0001**	0.82 (0.74, 0.92) ***p* = 0.0006**	1.51 (1.29, 1.77) ***p* < 0.0001**	1.64 (1.50, 1.79) ***p* < 0.0001**

Odds ratios (OR) and 95% confidence intervals (CI) generated using univariable logistic regression models. Bold values indicate statistical significance (*p* < 0.05). BMI, body mass index, NM, normotension; IDH, isolated diastolic hypertension; ISH, isolated systolic hypertension; SDH, systolic-diastolic hypertension; SBP, systolic blood pressure, DBP, diastolic blood pressure, FBG, fasting blood glucose, TG, triglyceride, TC, total cholesterol, LDL-C, low-density lipoprotein cholesterol, HDL-C, high-density lipoprotein cholesterol, TyG index, triglyceride-glucose index.

Table 3 presents the univariable and multivariable logistic regression analyses controlling for different predictor variables. In all 3 models, the TyG index showed a positive association with ISH, IDH, and SDH. Specifically, the odds of having hypertension were increased in the order of ISH, IDH, and SDH in Model 1. Model 2 suggested that increased odds of having IDH and SDH were associated with elevated TyG index controlling for other co-variables, but no statistically significant association was observed between the TyG index and ISH (OR = 0.98, 95% CI 0.69, 1.40). On the contrary, a decreased odds of being normotensive (e.g., no ISH, IDH, or SDH) was associated with an increased level of TyG index, and this association was statistically significant.

**TABLE 3:** Multivariate logistic regression analyses of the associations of increased TyG index and hypertension subtypes, Shandong, China (2007–2010).

	Model 0	Model 1	Model 2
	OR (95% CI), *P*-value	OR (95% CI), *P*-value	OR (95% CI), *P*-value
NM	0.43 (0.40, 0.48) ***p*** < **0.0001**	0.47 (0.42, 0.51) ***p*** < **0.0001**	0.54 (0.41, 0.71) ***p*** < **0.0001**
ISH	1.31 (1.17, 1.46) ***p*** < **0.0001**	1.20 (1.06, 1.35) ***p*** = **0.0033**	0.98 (0.69, 1.40) ***p*** = 0.9079
IDH	1.84 (1.55, 2.18) ***p*** < **0.0001**	1.91 (1.62, 2.26) ***p*** < **0.0001**	2.94 (1.66, 5.23) ***p*** = **0.0002**
SDH	2.06 (1.86, 2.27) ***p*** < **0.0001**	1.96 (1.77, 2.17) ***p*** < **0.0001**	1.82 (1.33, 2.49) ***p*** = **0.0002**

Model 0, unadjusted; Model 1: adjusted for age and sex only; Model 2: adjusted for age, sex, pulse, TC, TG, FBG, HDL-C, LDL-C, BMI, abdominal obesity, smoking history, and binge drinking. Odds ratios (OR) and 95% confidence intervals (CI) generated using univariable logistic regression model (for Model 0) and multivariable logistic regression models (for Model 1 and Model 2). Bold values indicated statistical significance (*p* < 0.05). NM, normotension; IDH, isolated diastolic hypertension; ISH isolated systolic hypertension; SDH systolic- diastolic hypertension; TyG index, triglyceride-glucose index.

### Trend between the predicted probabilities of having isolated systolic hypertension, isolated diastolic hypertension, systolic-diastolic hypertension, and triglyceride-glucose index

**FIGURE 1:**
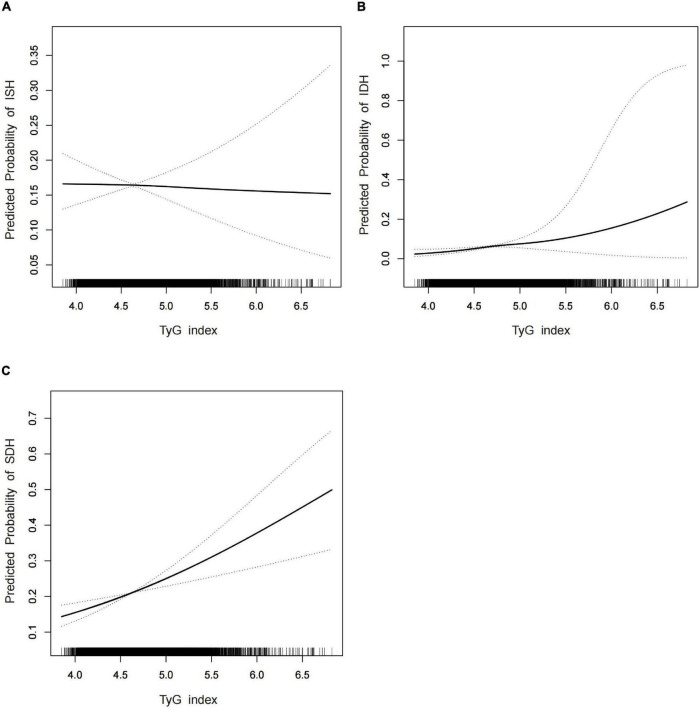
(A–C) The fitted smooth demonstrated the trend between the predicted probabilities of ISH and TyG index (A), the predicted probability of IDH and TyG index (B), and the predicted probability of SDH and TyG index (C). Adjusted factors included age, sex, pulse, TC, TG, FBG, HDL-C, LDL-C, BMI, abdominal obesity, smoking history, and binge drinking. The solid line and dashed line represent the estimated values and their corresponding 95% confidence intervals.

Loess fitted smooth curves captured the overall trend and patterns in the data while presenting the predicted probabilities of having ISH, IDH, SDH, and TyG index. To summarize, no obvious trend was found in terms of predicted probabilities of having ISH and TyG index (Figure 1A). Alternatively, Figure 1B illustrated an increasing, linear trend between the predicted probability of having IDH and TyG index, and a similar trend was found between the predicted probabilities of having SDH and TyG index (Figure 1C).

### Stratified analysis of the associations between the triglyceride-glucose index and isolated systolic hypertension, isolated diastolic hypertension, and systolic-diastolic hypertension

Stratified analyses were performed to further assess the associations between the TyG index and ISH, IDH, and SDH among different subgroups, and relevant results are presented in Table 4. Subgroup analyses revealed a highly consistent tendency. Of note, the effects of TyG index on hypertension subtypes were significant in females but not in males with respect to IDH (OR_females_ = 6.58, 95% CI: 2.29, 18.86 vs. OR_males_ = 1.77, 95% CI: 0.86, 3.65) and SDH (OR_females_ = 2.07, 95% CI: 1.30, 3.28 vs. OR_males_ = 1.49, 95% CI: 0.96, 2.32). Moreover, in the first quartile of age, a significantly positive association was observed between the TyG index and IDH (OR = 3.29, 95% CI:1.36, 7.96) and SDH (OR = 3.49, 95% CI: 1.61, 7.56), whereas no significant relationship was observed between the TyG index and ISH (OR = 0.73, 95% CI: 0.20, 2.59). Remarkably, among participants with lower lipid profiles and glucose levels, a higher OR magnitude was observed between the TyG index and IDH compared with the TyG index-SDH association. Ultimately, statistically significant 2-way interactions were found between TyG index and sex (*p* = 0.0374), TyG index and age (*p* = 0.0021), and TyG index and HDL-C (*p* = 0.0491) in the SDH group and between TyG index and BMI (*p* = 0.0443) in the ISH group.

**TABLE 4:** Stratified analyses of the associations between hypertension subtypes and TyG index with interactions, Shandong, China (2007−2010).

Variable with strata	N	ISH OR (95%CI)	*P*-value for interaction	IDH OR (95%CI)	*P*-value for interaction	SDH OR (95%CI)	*P*-value for interaction
Sex			0.9580		0.0762		**0.0374**
Male	6,444	0.84 (0.48, 1.44) *p* = 0.5214		1.77 (0.86, 3.65) *p* = 0.1197		1.49 (0.96, 2.32) *p* = 0.0762	
Female	10,349	0.97 (0.59, 1.59) *p* = 0.9052		6.58 (2.29, 18.86) ***p* = 0.0005**		2.07 (1.30, 3.28) ***p* = 0.0021**	
Age (years)			0.5313		0.1152		**0.0021**
Q1 (18−42)	4,160	0.73 (0.20, 2.59) *p* = 0.6251		3.29 (1.36, 7.96) ***p* = 0.0083**		3.49 (1.61, 7.56) ***p* = 0.0016**	
Q2 (43−52)	4,200	1.46 (0.59, 3.65) *p* = 0.4141		2.28 (0.88, 5.95) *p* = 0.0914		0.92 (0.48, 1.74) *p* = 0.7898	
Q3 (53−59)	4,081	1.68 (0.79, 3.57) *p* = 0.1783		5.84 (1.05, 32.48) ***p* = 0.0436**		1.62 (0.88, 2.97) *p* = 0.1219	
Q4 (60−75)	4,352	0.80 (0.47, 1.39) *p* = 0.4336		2.32 (0.25, 21.33) *p* = 0.4571		2.49 (1.31, 4.75) ***p* = 0.0056**	
Pulse (per 60 s)			0.3217		0.0683		0.7871
Q1 (55−67)	3,958	4.43 (1.61,12.22) ***p* = 0.0040**		13.22 (1.19, 146.60) ***p* = 0.0355**		1.50 (0.66, 3.39) *p* = 0.3329	
Q2 (68−73)	3,979	0.59 (0.28, 1.25) *p* = 0.1683		2.93 (0.64, 13.45) *p* = 0.1673		1.66 (0.85, 3.26) *p* = 0.1370	
Q3 (74−80)	4,371	0.86 (0.43, 1.71) *p* = 0.6602		2.72 (1.12, 6.62) ***p* = 0.0270**		2.22 (1.21, 4.08) ***p* = 0.0103**	
Q4 (81−112)	4,485	0.61 (0.32, 1.17) *p* = 0.1364		3.59 (1.23, 10.50) ***p* = 0.0196**		1.82 (1.04, 3.17) ***p* = 0.0345**	
TC (mmol/L)			0.9176		0.0734		0.0760
Q1 (1.2−4.0)	4,192	0.93 (0.40, 2.15) *p* = 0.8699		3.00 (1.04, 8.70) ***p* = 0.0426**		2.56 (1.14, 5.75) ***p* = 0.0230**	
Q2 (4.0−4.6)	4,134	1.48 (0.53, 4.12) *p* = 0.4533		38.10 (3.89, 373.54) ***p* = 0.0018**		1.41 (0.69, 2.87) *p* = 0.3422	
Q3 (4.6−5.2)	4,233	1.73 (0.64, 4.65) *p* = 0.2763		3.01 (0.69, 13.03) *p* = 0.1414		1.75 (0.83, 3.65) *p* = 0.1394	
Q4 (5.2−15.9)	4,234	0.84 (0.47, 1.52) *p* = 0.5683		1.48 (0.59, 3.76) *p* = 0.4044		1.52 (0.88, 2.62) *p* = 0.1290	
TG (mmol/L)			0.9039		0.2383		0.7248
Q1 (0.2−0.8)	4,083	0.74 (0.21, 2.65) *p* = 0.6432		4.64 (0.48, 45.09) *p* = 0.1859		3.69 (1.04,13.06) ***p* = 0.0430**	
Q2 (0.8−1.1)	4,220	1.24 (0.22, 6.82) *p* = 0.8064		3.14 (0.16, 62.89) 0 *p* = 0.4545		0.44 (0.09, 2.12) *p* = 0.3045	
Q3 (1.1−1.7)	4,282	1.04 (0.29, 3.69) *p* = 0.9562		1.57 (0.24, 10.46) *p* = 0.6407		1.94 (0.61, 6.22) *p* = 0.2640	
Q4 (1.7−19.4)	4,208	1.35 (0.99, 1.84) *p* = 0.0599		1.04 (0.68, 1.57) *p* = 0.8707		1.20 (0.93, 1.55) *p* = 0.1556	
FBG (mmol/L)			0.2804		0.8932		0.1565
Q1 (3.3−4.3)	4,148	0.61 (0.26, 1.39) *p* = 0.2361		2.31 (0.57, 9.37) *p* = 0.2421		3.15 (1.35, 7.39) ***p* = 0.0082**	
Q2 (4.3−4.7)	4,203	0.49 (0.21, 1.17) *p* = 0.1067		3.44 (0.75, 15.85) *p* = 0.1127		1.66 (0.71, 3.89) *p* = 0.2470	
Q3 (4.7−5.3)	4,243	1.50 (0.63, 3.53) *p* = 0.3579		1.59 (0.56, 4.51) *p* = 0.3835		2.40 (1.24, 4.65) ***p* = 0.0095**	
Q4 (5.3−19.1)	4,199	1.14 (0.71, 1.83) *p* = 0.5897		1.88 (0.92, 3.84) *p* = 0.0828		1.09 (0.72, 1.65) *p* = 0.6756	
HDL-C (mmol/L)			0.7500		0.5671		**0.0491**
Q1 (0.0−1.3)	4,144	1.11 (0.55, 2.26) *p* = 0.7726		5.98 (1.44, 24.80) ***p* = 0.0137**		1.58 (0.86, 2.90) *p* = 0.1420	
Q2 (1.3−1.5)	4,252	1.44 (0.62, 3.34) *p* = 0.3961		5.92 (1.60, 21.94) ***p* = 0.0078**		1.76 (0.93, 3.35) *p* = 0.0848	
Q3 (1.5−1.8)	4,084	2.15 (0.72, 6.46) *p* = 0.1708		2.62 (0.51, 13.40) *p* = 0.2468		1.14 (0.51, 2.55) *p* = 0.7485	
Q4 (1.8−3.8)	4,313	0.74 (0.41, 1.32) *p* = 0.3044		1.53 (0.61, 3.85) *p* = 0.3673		1.99 (1.10, 3.61) ***p* = 0.0230**	
LDL-C (mmol/L)			0.8761		0.0698		0.7045
Q1 (0.0−2.7)	4,181	1.36 (0.52, 3.54) *p* = 0.5323		3.46 (1.19, 10.02) ***p* = 0.0222**		2.21 (1.07, 4.55) ***p* = 0.0324**	
Q2 (2.7−3.0)	4,106	0.72 (0.28, 1.89) *p* = 0.5051		19.01 (2.46, 146.64) ***p* = 0.0047**		2.05 (0.75, 5.55) *p* = 0.1592	
Q3 (3.0−3.4)	4,257	1.70 (0.59, 4.90) *p* = 0.3280		2.90 (0.63, 13.30) *p* = 0.1716		1.47 (0.71, 3.05) *p* = 0.3021	
Q4 (3.4−9.7)	4,249	0.89 (0.49, 1.61) *p* = 0.7003		1.29 (0.52, 3.18) *p* = 0.5803		1.75 (1.02, 2.99) ***p* = 0.0404**	
BMI (kg/m^2^)			**0.0443**		0.8472		0.8613
Q1 (13.3−21.7)	4,196	1.68 (0.60, 4.68) *p* = 0.3209		10.88 (0.94, 125.37) *p* = 0.0557		2.62 (1.03, 6.67) ***p* = 0.0428**	
Q2 (21.7−24.1)	4,198	1.30 (0.64, 2.62) *p* = 0.4660		3.59 (0.66, 19.47) *p* = 0.1387		2.12 (1.06, 4.24) ***p* = 0.0347**	
Q3 (24.1−26.6)	4,200	0.44 (0.22, 0.86) ***p* = 0.0163**		3.09 (1.02, 9.38) ***p* = 0.0469**		1.98 (1.06, 3.69) ***p* = 0.0315**	
Q4 (26.6−40.9)	4,199	1.15 (0.58, 2.29) *p* = 0.6886		2.52 (1.08, 5.85) ***p* = 0.0317**		1.73 (1.03, 2.90) ***p* = 0.0384**	

Odds ratios (OR) and 95% confidence intervals (CI) generated using multivariable logistic regression models. Bold values indicate statistical significance (*p* < 0.05). BMI, body mass index, NM, normotension; IDH, isolated diastolic hypertension; ISH, isolated systolic hypertension; SDH, systolic-diastolic hypertension; FBG, fasting blood glucose; TG, triglyceride; TC, total cholesterol; LDL-C, low-density lipoprotein cholesterol; HDL-C, high-density lipoprotein cholesterol; TyG index, triglyceride-glucose index. All confounding variables (age, sex, pulse, TC, TG, FBG, HDL-C, LDL-C, BMI, abdominal obesity, smoking history, binge drinking) were adjusted for each stratification, except the stratification factor itself.

## Discussion

In this cross-sectional study, we investigated the associations of the TyG index with different hypertension subtypes in a sample of 16,793 Chinese adults from Shandong Province. Multivariate logistic regression analyses revealed significant associations between the TyG index and IDH and between the TyG index and SDH. Interestingly, there was no significant relationship between the TyG index and ISH, and this finding might be explained by a lower effect of glycolipid metabolic disorder (e.g., IR) on ISH.

It is well known that IR serves as one of the most significant risk factors for developing hypertension (23). However, the underlying mechanisms have not been fully elucidated. Possible mechanisms could be related to the following aspects: First, IR can stimulate the activity of the renin-angiotensin-aldosterone system (RAAS), which further promotes salt absorption and water–sodium retention, and ultimately leads to hypertension (24). Second, hyperinsulinemia activates the sympathetic nervous system and increases cardiac output and peripheral vascular resistance (25). The secretion of catecholamine may result in the hypertrophy of vascular smooth muscle cells and endothelial cells, leading to luminal stenosis (26). Finally, insulin may exert an important role in the contraction and vasodilatation of the peripheral vasculature by the release of endothelin, which may contract the blood vessels, and decrease the synthesis of prostacyclin (PGI2) and prostaglandin E2 (PGE2), factors that may dilate vessels (27), ultimately leading to BP elevation. In this study, abnormal glycolipid metabolism and IR may have more impact on IDH, and one possible explanation is that a higher FBG and abnormal lipid panel may increase the blood viscosity by increasing the rheological component of peripheral resistance, leading to elevated DBP (28). Meanwhile, overweight and obesity, which is related to excessive accumulation of adipose tissue in the outer peripheral vascular, may further restrict vasomotor of peripheral vascular and cause IDH (29). This is consistent with our results showing that the TyG index is more closely associated with IDH than with ISH. Further experiments need to be conducted to better elucidate the mechanisms of how IR could potentially affect IDH.

Several studies have reported that an elevated TyG index has been suggested as a risk factor for hypertension (30, 31). A 9-year longitudinal population-based study demonstrated that a higher TyG index could predict a higher incidence of hypertension in Chinese people; however, this study only focused on Chinese males and no stratified analysis was performed (16). Another cross-sectional study was conducted in children and adolescents, which stated that an elevated TyG index was significantly associated with a higher prevalence of pre-hypertension and hypertension (32). Compared with a previous study that also investigated the associations between TyG index and hypertension (33), the major strength of our study is that it is the first study to examine the associations between the TyG index and different hypertension subtypes. In addition, a population-based study with a large sample size, which potentially allows a more robust analysis with better generalizability, is also a strength.

In this study, stratified analyses suggested a significant association between the TyG index and hypertension in women but not in men. A recent study claimed that the TyG index was associated with carotid atherosclerosis and arterial stiffness, and of note, carotid atherosclerosis and arterial stiffness were two conditions that were found mostly in lean, postmenopausal women (34). Relatedly, TyG was also associated with an increased odds of pre-hypertension in lean females but not in males (35). Moreover, this study also suggested a significant interaction between the TyG index and sex in the SDH group. The cause of the gender disparity could be attributed to some gender-specific differences in glycolipid metabolism, adipose distribution, IR, and the effect of estrogen (36, 37). Therefore, clinicians should pay special attention to women with an increased TyG index, and gender-specific cut-off values should be established.

Previous studies have indicated that people with IDH have higher odds of converting IDH to SDH in 10 years compared with people without hypertension, and this finding was significant in young adults (38, 39). Interestingly, from the stratified analyses, we found that the TyG index in younger participants (Q1) was significantly associated with IDH and SDH. The interaction between the TyG index and age was also significant in the SDH group. Our result was consistent with the literature, which suggested that compared with older patients, younger patients had a higher prevalence of IDH but a lower prevalence of ISH (40). This finding can be possibly attributed to the effect of abnormal glycolipid metabolism with increased peripheral vascular resistance rather than arterial stiffness within a short period of time in younger populations, which strengthens the evidence that abnormal glycolipid metabolism may have little influence on SBP among younger people. Another study reported that among 1,777 participants aged 40 years and older, a significant association between TyG index and increased risk of ISH was found, yet, no in the TyG index–IDH association (41). However, given that the aforementioned study was conducted in middle aged and elderly adults with only 85 IDH patients, the lack of associations may be due to the relatively small sample size and the age group of interest. Currently, awareness, treatment, and control of hypertension are relatively low among young and middle-aged adults with IDH, and the importance of hypertension screening in young and middle-aged adults with an elevated TyG index should be highlighted.

Two-way interaction analyses were performed to better illustrate the associations of TyG index and hypertension, and statistically significant effect modifications were observed between TyG index and age in the SDH group, TyG index and sex in the SDH group, TyG index and HDL-C in the SDH group, and TyG index and BMI in the ISH group. To our knowledge, very limited study has examined the potential effect modification with a focus on the TyG index in hypertension. One hypertension study suggested that several additive interactions were found between the TyG index and certain anthropometric measures such as waist-to-height ratio and percentage of body fat only (33). Alternatively, Tang et al. (42) demonstrated a potentiating effect modification between high BMI (e.g., overweight and obesity) and hypercholesteremia on hypertension. The observed interaction between BMI and hyperlipidemia can be potentially explained by an increased risk of developing metabolic syndrome and IR among overweight and obese people, which subsequently leads to the development of hypertension (43). Further study is needed to better understand the interactions between the TyG index and other key risk factors of hypertension.

This study has several limitations that should be noted. First, because no insulin level test was performed, we were unable to measure the degree of IR, which was also considered as a potential risk factor for hypertension. Second, while a large, population-based study was conducted, only participants from the Shandong province were recruited and included in our analysis, which may or may not be generalizable to the whole population in China. Finally, the cross-sectional study design can only show whether an association exists between the TyG index and a hypertension subtype, not causation. Hence, large longitudinal studies should be carried out to validate if a causal relationship exists between the TyG index and a specific hypertension subtype.

## Conclusion

This study has further revealed the significant relationships between TyG index and different hypertension subtypes in a large population-based study in China, suggesting that an increased TyG index was significantly associated with IDH and SDH. In contrast, no significant association was observed between the TyG index and ISH. From a clinical perspective, the TyG index can be utilized as a novel indicator for IDH and SDH, and our findings might also inform the development and implementation of targeted screening for hypertension.

## Data availability statement

The original contributions presented in this study are included in the article/supplementary material, further inquiries can be directed to the corresponding author/s.

## Ethics statement

The studies involving human participants were reviewed and approved by the Shandong First Medical University and Shandong Academy of Medical Sciences. The patients/participants provided their written informed consent to participate in this study.

## Author contributions

QC and CX analyzed the data and wrote the initial draft of the manuscript. JZ and YW recruited the subjects and supervised the study. FL and JP contributed to the conception and design of the study. CX and JP contributed to the writing, reviewing, and revising of the manuscript. JP took responsibility for all aspects of the reliability and freedom from bias of the data presented and their discussed interpretation. All authors have read and approved the final manuscript.

## Footnotes

1
http://www.r-project.org

2
www.empowerstats.com

## Abbreviations

CVD: cardiovascular disease
ISH: isolated systolic hypertension
IDH: isolated diastolic hypertension
SDH: systolic-diastolic hypertension
NM: normotension
BP: blood pressure
DBP: diastolic blood pressure
SBP: systolic blood pressure
BG: blood glucose
TG: triglyceride
TC: total cholesterol
LDL-C: low-density lipoprotein cholesterol
HDL-C: high-density lipoprotein cholesterol
TyG index: triglyceride-glucose index
FBG: fasting blood glucose
BMI: body mass index
SD: standard deviation
OR: odds ratio.

## References

[1] Jones DW Whelton PK Allen N Clark D III Gidding SS Muntner P Management of Stage 1 hypertension in adults with a low 10-year risk for cardiovascular disease: Filling a guidance gap: A scientific statement from the American Heart Association. *Hypertension.* (2021) 77:e58–67. 10.1161/HYP.0000000000000195 33910363

[2] Gao Y Chen G Tian H Lin L Lu J Weng J Prevalence of hypertension in china: A cross-sectional study. *PLoS One.* (2013) 8:e65938. 10.1371/journal.pone.0065938 23776574 PMC3679057

[3] Li Q Liu C Zhang S Li R Zhang Y He P Dietary carbohydrate intake and new-onset hypertension: A nationwide cohort study in China. *Hypertension.* (2021) 78:422–30. 10.1161/HYPERTENSIONAHA.120.16751 33550823

[4] Wang Z Chen Z Zhang L Wang X Hao G Zhang Z Status of hypertension in China: Results from the China hypertension survey, 2012-2015. *Circulation.* (2018) 137:2344–56. 10.1161/CIRCULATIONAHA.117.032380 29449338

[5] Ghatage T Goyal SG Dhar A Bhat A. Novel therapeutics for the treatment of hypertension and its associated complications: Peptide- and nonpeptide-based strategies. *Hypertens Res.* (2021) 44:740–55. 10.1038/s41440-021-00643-z 33731923 PMC7967108

[6] Lee H Yano Y Cho SMJ Park JH Park S Lloyd-Jones DM Cardiovascular risk of isolated systolic or diastolic hypertension in young adults. *Circulation.* (2020) 141:1778–86. 10.1161/CIRCULATIONAHA.119.044838 32479205

[7] Asgari S Khalili D Mehrabi Y Kazempour-Ardebili S Azizi F Hadaegh F. Incidence and risk factors of isolated systolic and diastolic hypertension: A 10 year follow-up of the Tehran Lipids and Glucose Study. *Blood Press.* (2016) 25:177–83. 10.3109/08037051.2015.1116221 26643588

[8] Lee JW Lim NK Baek TH Park SH Park HY. Anthropometric indices as predictors of hypertension among men and women aged 40-69 years in the Korean population: The Korean genome and epidemiology study. *BMC Public Health.* (2015) 15:140. 10.1186/s12889-015-1471-5 25886025 PMC4332746

[9] Sheriff HM Tsimploulis A Valentova M Anker MS Deedwania P Banach M Isolated diastolic hypertension and incident heart failure in community-dwelling older adults: Insights from the Cardiovascular Health Study. *Int J Cardiol.* (2017) 238:140–3. 10.1016/j.ijcard.2017.02.142 28343761 PMC6454920

[10] Yi Q Zha M Yang Q Zhang Y Hou L Ye X Trends in the prevalence of hypertension according to severity and phenotype in Chinese adults over two decades (1991-2015). *J Clin Hypertens (Greenwich).* (2021) 23:1302–15. 10.1111/jch.14306 34128308 PMC8678778

[11] O’Meara JG Kardia SL Armon JJ Brown CA Boerwinkle E Turner ST. Ethnic and sex differences in the prevalence, treatment, and control of dyslipidemia among hypertensive adults in the GENOA study. *Arch Intern Med.* (2004) 164:1313–8. 10.1001/archinte.164.12.1313 15226165

[12] Janghorbani M Bonnet F Amini M. Glucose and the risk of hypertension in first-degree relatives of patients with type 2 diabetes. *Hypertens Res.* (2015) 38:349–54. 10.1038/hr.2015.10 25693857

[13] Gouaref I Bouazza A Abderrhmane SA Koceir EA. Lipid profile modulates cardiometabolic risk biomarkers including hypertension in people with Type-2 diabetes: A focus on unbalanced ratio of plasma polyunsaturated/saturated fatty acids. *Molecules.* (2020) 25:4315. 10.3390/molecules25184315 32962299 PMC7570813

[14] Nordestgaard BG Varbo A. Triglycerides and cardiovascular disease. *Lancet.* (2014) 384:626–35. 10.1016/S0140-6736(14)61177-6 25131982

[15] Antonio-Villa NE Bello-Chavolla OY Vargas-Vazquez A Mehta R Fermin-Martinez CA Martagon-Rosado AJ Increased visceral fat accumulation modifies the effect of insulin resistance on arterial stiffness and hypertension risk. *Nutr Metab Cardiovasc Dis.* (2021) 31:506–17. 10.1016/j.numecd.2020.09.031 33279372

[16] Zheng R Mao Y. Triglyceride and glucose (TyG) index as a predictor of incident hypertension: A 9-year longitudinal population-based study. *Lipids Health Dis.* (2017) 16:175. 10.1186/s12944-017-0562-y 28903774 PMC5598027

[17] Spence JD Huff M Barnett PA. Effects of indapamide versus hydrochlorothiazide on plasma lipids and lipoproteins in hypertensive patients: A direct comparison. *Can J Clin Pharmacol.* (2000) 7:32–7. 10822211

[18] Group NS McMurray JJ Holman RR Haffner SM Bethel MA Holzhauer B Effect of valsartan on the incidence of diabetes and cardiovascular events. *N Engl J Med.* (2010) 362:1477–90. 10.1056/NEJMoa1001121 20228403

[19] Centers for Disease Control and Prevention [CDC]. *National center for health statistics – National health interview survey, glossary.* (2022). Available online at: https://www.cdc.gov/nchs/nhis/tobacco/tobacco_glossary.htm (accessed June 15, 2022).

[20] Centers for Disease Control and Prevention [CDC]. *National center for health statistics – National health interview survey, glossary.* (2022). Available online at: https://www.cdc.gov/nchs/nhis/alcohol/alcohol_glossary.htm (accessed June 15, 2022).

[21] Simental-Mendia LE Rodriguez-Moran M Guerrero-Romero F. The product of fasting glucose and triglycerides as surrogate for identifying insulin resistance in apparently healthy subjects. *Metab Syndr Relat Disord.* (2008) 6:299–304. 10.1089/met.2008.0034 19067533

[22] Williams B Mancia G Spiering W Rosei EA Azizi M Burnier M [2018 ESC/ESH Guidelines for the management of arterial hypertension]. *Kardiol Pol.* (2019) 77:71–159. 10.5603/KP.2019.0018 30816983

[23] Lytsy P Ingelsson E Lind L Arnlov J Sundstrom J. Interplay of overweight and insulin resistance on hypertension development. *J Hypertens.* (2014) 32:834–9. 10.1097/HJH.0000000000000081 24370898

[24] Soleimani M. Insulin resistance and hypertension: New insights. *Kidney Int.* (2015) 87:497–9. 10.1038/ki.2014.392 25723632

[25] da Silva AA do Carmo JM Li X Wang Z Mouton AJ Hall JE. Role of hyperinsulinemia and insulin resistance in hypertension: Metabolic syndrome revisited. *Can J Cardiol.* (2020) 36:671–82. 10.1016/j.cjca.2020.02.066 32389340 PMC7219403

[26] Shin E Ko KS Rhee BD Han J Kim N. Different effects of prolonged beta-adrenergic stimulation on heart and cerebral artery. *Integr Med Res.* (2014) 3:204–10. 10.1016/j.imr.2014.10.002 28664099 PMC5481746

[27] Muniyappa R Chen H Montagnani M Sherman A Quon MJ. Endothelial dysfunction due to selective insulin resistance in vascular endothelium: Insights from mechanistic modeling. *Am J Physiol Endocrinol Metab.* (2020) 319:E629–46. 10.1152/ajpendo.00247.2020 32776829 PMC7642854

[28] Zhang R Reisin E. Obesity-hypertension: The effects on cardiovascular and renal systems. *Am J Hypertens.* (2000) 13:1308–14. 10.1016/s0895-7061(00)01254-1 11130776

[29] Grebla RC Rodriguez CJ Borrell LN Pickering TG. Prevalence and determinants of isolated systolic hypertension among young adults: The 1999-2004 US National Health And Nutrition Examination Survey. *J Hypertens.* (2010) 28:15–23. 10.1097/HJH.0b013e328331b7ff 19730124 PMC2891994

[30] Zhu B Wang J Chen K Yan W Wang A Wang W A high triglyceride glucose index is more closely associated with hypertension than lipid or glycemic parameters in elderly individuals: A cross-sectional survey from the Reaction Study. *Cardiovasc Diabetol.* (2020) 19:112. 10.1186/s12933-020-01077-6 32664945 PMC7362407

[31] Zhang F Zhang Y Guo Z Yang H Ren M Xing X The association of triglyceride and glucose index, and triglyceride to high-density lipoprotein cholesterol ratio with prehypertension and hypertension in normoglycemic subjects: A large cross-sectional population study. *J Clin Hypertens (Greenwich).* (2021) 23:1405–12. 10.1111/jch.14305 34118112 PMC8678664

[32] Simental-Mendia LE Hernandez-Ronquillo G Gamboa-Gomez CI Gomez-Diaz R Rodriguez-Moran M Guerrero-Romero F. The triglycerides and glucose index is associated with elevated blood pressure in apparently healthy children and adolescents. *Eur J Pediatr.* (2019) 178:1069–74. 10.1007/s00431-019-03392-x 31081518

[33] Wang K He G Zhang Y Yin J Yan Y Zhang Y Association of triglyceride-glucose index and its interaction with obesity on hypertension risk in Chinese: A population-based study. *J Hum Hypertens.* (2021) 35:232–9. 10.1038/s41371-020-0326-4 32203074

[34] Lambrinoudaki I Kazani MV Armeni E Georgiopoulos G Tampakis K Rizos D The TyG index as a marker of subclinical atherosclerosis and arterial stiffness in lean and overweight postmenopausal women. *Heart Lung Circ.* (2018) 27:716–24. 10.1016/j.hlc.2017.05.142 28690023

[35] Zeng ZY Liu SX Xu H Xu X Liu XZ Zhao XX. Association of triglyceride glucose index and its combination of obesity indices with prehypertension in lean individuals: A cross-sectional study of Chinese adults. *J Clin Hypertens (Greenwich).* (2020) 22:1025–32. 10.1111/jch.13878 32442359 PMC8029919

[36] Tramunt B Smati S Grandgeorge N Lenfant F Arnal JF Montagner A Sex differences in metabolic regulation and diabetes susceptibility. *Diabetologia.* (2020) 63:453–61. 10.1007/s00125-019-05040-3 31754750 PMC6997275

[37] Di Giosia P Giorgini P Stamerra CA Petrarca M Ferri C Sahebkar A. Gender differences in epidemiology, pathophysiology, and treatment of hypertension. *Curr Atheroscler Rep.* (2018) 20:13. 10.1007/s11883-018-0716-z 29445908

[38] Franklin SS Pio JR Wong ND Larson MG Leip EP Vasan RS Predictors of new-onset diastolic and systolic hypertension: The Framingham Heart Study. *Circulation.* (2005) 111:1121–7. 10.1161/01.CIR.0000157159.39889.EC 15723980

[39] Saladini F Dorigatti F Santonastaso M Mos L Ragazzo F Bortolazzi A Natural history of hypertension subtypes in young and middle-age adults. *Am J Hypertens.* (2009) 22:531–7. 10.1038/ajh.2009.21 19229194

[40] Bavishi C Goel S Messerli FH. Isolated systolic hypertension: An update after SPRINT. *Am J Med.* (2016) 129:1251–8. 10.1016/j.amjmed.2016.08.032 27639873

[41] Jian S Su-Mei N Xue C Jie Z Xue-Sen W. Association and interaction between triglyceride-glucose index and obesity on risk of hypertension in middle-aged and elderly adults. *Clin Exp Hypertens.* (2017) 39:732–9. 10.1080/10641963.2017.1324477 28737433

[42] Tang N Ma J Tao R Chen Z Yang Y He Q The effects of the interaction between BMI and dyslipidemia on hypertension in adults. *Sci Rep.* (2022) 12:927. 10.1038/s41598-022-04968-8 35042940 PMC8766602

[43] Rao A Pandya V Whaley-Connell A. Obesity and insulin resistance in resistant hypertension: Implications for the kidney. *Adv Chronic Kidney Dis.* (2015) 22:211–7. 10.1053/j.ackd.2014.12.004 25908470

